# Editorial: Lipids in host and protozoan parasite interaction

**DOI:** 10.3389/fcimb.2023.1334002

**Published:** 2023-11-17

**Authors:** Silvia Haase, Lesly Temesvari, Kai Zhang

**Affiliations:** ^1^The Francis Crick Institute, London, United Kingdom; ^2^Department of Biological Sciences, Clemson University, Clemson, SC, United States; ^3^Department of Biological Sciences, Texas Tech University, Lubbock, TX, United States

**Keywords:** lipid metabolism, membrane trafficking, cell signaling, lipid droplets, lipid rafts, parasites

Protozoan parasites have developed remarkable strategies to thrive in their respective biological niches. With this Research Topic we aimed to draw more attention to the importance of lipid biology in the establishment of infection and parasite survival. While lipids form a large and essential class of biomolecules, their role in host and parasite interactions is poorly understood. Identifying the molecular mechanisms by which Protozoa employ lipids to promote their pathogenicity may provide novel concepts towards the development of antiparasitic drugs.

Malaria parasites require vast amounts of suitable lipids to proliferate and maintain membrane homeostasis throughout their complex lifecycle. To meet this high demand under highly diverse conditions, *Plasmodium* spp. scavenges lipids and precursors from its host and deploys *de novo* lipid synthesis to ensure parasite development. While much is known about the lipid biosynthesis pathways in *Plasmodium* spp., the proteins involved in lipid acquisition, trafficking and repurposing remain to be unravelled. Shunmugam et al. identified a putative patatin-like phospholipase in *P. falciparum*, *Pf*PNPLA2, as a novel molecular player in recycling parasite lipids. The authors investigate its expression and localisation and utilise a conditional knockdown approach showing the detrimental effect of losing *Pf*PNPLA2 function under lipid-limiting conditions. A lipidomic analysis reveals blood stage-specific differences in the synthesis of neutral lipids, an impact on overall phospholipid homeostasis, as well as significant accumulation of phosphatidylglycerol and decrease of lysobisphosphatidic acid in *Pf*PNPLA2-deficient parasites. Knockdown of the phospholipase also leads to increased accumulation of storage lipids and free fatty acids (FAs), pointing towards an increase of nutrient uptake from the host. Collectively, their data suggest a compensating mechanism by which *Pf*PNPLA2 degrades PG to generate LBPA and acquire free FAs to ensure lipid synthesis and therefore parasite survival when host nutrient resources are low.

Kinetoplastid parasites utilise multiple lipid synthesis processes that are either absent or different from those in the human host. Cerone et al. combined genome mining, lipidomic analysis, and experimental manipulation of cultures to present a detailed characterization of lipid biosynthesis in *Crithidia fasciculata*. Although not infective to humans, *C. fasciculata* has served as a model for infectious kinetoplastids including *Trypanosoma brucei*, *T. cruzi*, and *Leishmania* spp. The ease with which it can be grown and the similarity of its metabolic pathways to those of the pathogenic kinetoplastids makes *C. fasciculata* a versatile model system. The authors demonstrate that *C. fasciculata* can synthesize all the main phospholipid species expected in eukaryotes. In addition, *C. fasciculata* can also incorporate extracellular FAs and possesses an unusual cyclopropyl FA typically found in prokaryotes. The uniqueness of this FA and its biosynthetic route may exemplify a viable target for drug discovery in kinetoplastids.

The review by Poudyal and Paul addressed the possible mechanisms *Trypanosoma brucei* may use to acquire FAs. The authors summarize the availability of FAs in various host niches and compare the uptake machineries between *T. brucei* and other eukaryotes. *T. brucei* can also synthesize FAs *de novo* in the cytosol and mitochondria. The flexibility in lipid acquisition likely contributes to the adaptability and persistence of *T. brucei* in mammalian hosts. Future work may reveal whether FAs from *de novo* synthesis and uptake contribute to different pools in *T. brucei* biology and explore the possibility of targeting FA scavenge for therapeutic purposes.

The study by Manzano et al. examined the impact of *Leishmania infantum* infection on cholesterol homeostasis in human macrophages which are the definitive host cells for *Leishmania* parasites. Infection of THP-1 cells with *L. infantum* clinical isolates that failed to respond to liposomal amphotericin B treatment led to a significant increase in plasma membrane cholesterol content and membrane rigidity. RNA-seq analyses of infected THP-1 cells suggest that the elevated level of membrane cholesterol can be attributed to increase in cholesterol biosynthesis/transport to the plasma membrane and defect in the transfer of cholesterol from the plasma membrane to the apolipoprotein particles. These findings differ from previous reports showing a reduction in macrophage membrane cholesterol and rigidity upon *Leishmania* infection, which could be due to differences in parasite species and macrophage types, or the timing of infection. Regardless, *Leishmania*-induced alteration of host cholesterol homeostasis may significantly compromise multiple processes such as IFNγ signalling and antigen presentation.

Cholesterol has also been the focus of the in-depth review by Maier & van Ooij detailing the intricate relationship between malaria parasites and this essential lipid. *Plasmodium* spp. are incapable of synthesizing cholesterol *de novo* but are highly dependent on it. The authors describe the changes in the cholesterol to phospholipid ratio within the plasma membrane of infected red blood cells (RBCs) and discuss possible uptake mechanisms and candidates. They then summarise the importance of cholesterol for the successful invasion of and development within hepatocytes and RBCs, its role in making gametocytes more transmissible and lastly the challenges the parasite faces in the mosquito, which (similar to RBCs) are unable to produce this key molecule.

Infections by parasites may also be facilitated by specialized membrane domains known as lipid rafts. These are detergent-resistant nanoscale assemblages of cholesterol, sphingolipids, and proteins, which function in signal transduction, the formation of extracellular vesicles, and cell-cell interactions in most Eukarya. Thus, it is of no surprise that lipid rafts on the surface of the enteric parasite *Giardia* facilitate parasite-host interaction. Adhesion of *Giardia* to the host intestinal epithelium is an indispensable virulence function. Grajeda et al. have carried out the most comprehensive characterization of lipid rafts in *Giardia* to date. Using direct stochastic-optical microscopy (dSTORM), they report that giardial lipid rafts are approximately 20 nm, which is among the smallest of lipid rafts in eukaryotes. The authors also inhibited attachment of *Giardia* to host cells *in vitro* by repurposing two raft-disrupting, FDA-approved drugs, nystatin and oseltamivir. Repurposing drugs for neglected diseases, such as giardiasis, is a popular strategy in drug discovery. Finally, the authors perform the first proteomic analysis of lipid rafts and raft-dependent extracellular vesicles of *Giardia*. Together, the findings set the stage for adopting raft-disrupting drugs to treat giardiasis.

## Author contributions

SH: Writing – original draft, Writing – review & editing. LT: Writing – original draft, Writing – review & editing. KZ: Writing – original draft, Writing – review & editing.

